# Abstract of the Reports of the Sick and Wounded at Buffalo Barracks, from 1st July 1842, to 30th June 1845—3 Years—by R. C. Wood, M. D., Surgeon U. S. Army

**Published:** 1845-11-01

**Authors:** E. R. Long

**Affiliations:** U. S. A.


### Abstract of the Reports

*of the Sick and Wounded at Buffalo Barracks, from 1st July 1842, to 30th June 1845—3 years—by R. C. Wood, M. D., Surgeon U. S. Army.*

Communicated for the Buffalo Medical Journal, by Lieut. E. R. LONG, M. D., U. S. A.

It is due to the contributor of the following article, to state that it was submitted for the Journal in a tabular form, prepared with much care and labor. It was found, however, that it could not be presented to our readers in this form without being extended over several pages, which would impair its appearance, and render it inconvenient for easy reference. We have deemed it best, therefore, to give the facts a different arrangement, in order to render them more readily accessible, and for the sake of greater condensation. Our thanks are due to our esteemed friend and correspondent for his valuable communication. We hope to hear from him frequently. The classification of diseases followed in this abstract is that prescribed by the regulations of the Medical Department of the United States Army.—EDITOR.

FEVERS.

*Continued.*—1842, in August, 1 case. 1844, March, 2 cases. April, 1 case. November, 1 case. Total for the three years, 5 cases.

*Intermittent.*—1842, July, 47 cases. August, 25 cases. September, 13 cases. October, 10 cases. November, 11 cases. December, 12 cases.—1843, January, 6 cases. February, 4 cases. March, 3 cases. April, 1 case. May, 4 cases. June, 1 case. July, 2 cases. August, 1 case. September, 1 case. October, 4 cases. November, 1 case. December, 1 case. 1844, May, 1 case. June, 5 cases. September, 4 cases. November, 6 cases. December, 1 case. Total number of cases, 168.

*Remittent or Bilious.*—Two cases only enter into the report, which occurred September, 1844.

*Ephemeral.*—Under this head 6 cases are reported, all of which occurred in October, 1843.

ERUPTIVE FEVERS.

*Erysipelas* —In June, 1843, 2 cases.

*Rubeola.*—In February, 1844, 1 case. In May, 1845, 6 cases. in June, 1 case. Total number, 8.

*Scarlatina.*—In April, 1845, 1 case.

DISEASES OF THE ORGANS CONNECTED WITH DIGESTION.

*Cholera.*—1843, April, 1 case. May, 1 case. July, 2 cases. August, 1 case. November, 1 case. 1844, October, 1 case. November, 1 case. Total number, 8.

*Colica.*—1842, September, 1 case. 1844, January, 1 case. June, 1 case. 1845, June, 2 cases. Total number, 5.

*Diarrhœa.*—1842, July, 27 cases. August, 19 cases. September, 13 cases. October, 2 cases. November, 2 cases. December, 5 cases. 1845, January, 3 cases. March, 3 cases. April, 2 cases. May, 3 cases. June, 2 cases. July, 16 cases. August, 18 cases. September, 4 cases. December, 3 cases. 1844, January, 1 case. February, 1 case. March, 2 cases. June, 3 cases. July, 24 cases. August, 13 cases. September, 11 cases. October, 2 cases. November, 2 cases. December, 1 case. 1815, January, 2 cases. February, 1 case. March, 2 cases. April, 2 cases. May, 6 cases. June, 6 cases. Total number, 201.

*Dysenteria Acuta.*—1844, June, 1 case. 1845, January, 1 case.—Total number, 2.

*Dysenteria Chronica.*—1842, November, 1 case. December, 1 case. 1844, June, 1 case. November, 1 case. Total number, 4.

*Gastritis.*—1843, August, 1 case. September, 1 case. Total number, 2.

*Hepatitis Chronica.*—1844, June, 1 case. August, 1 case. Total number, 2.

*Icterus.*—1843, December, 1 case. 1844, June, 1 case. August, 1 case Total number, 3.

*Obstipatio.*—1842, July, 9 cases. September, 1 case. November, 1 case. December, 2 cases. 1843, January, 1 case. February, 1 case.—April, 1 case. June, 1 case. July, 1 case. August, 1 case. September, 1 case. 1844, August, 1 case. September, 1 case. November, 1 case.—April, 1 case. 1845, May, 2 cases. June, 1 case. Total number, 27.

*Cynanche Parotidea.*—1844, February, 1 case. March, 5 cases. July 1 case. August, 1 case. Total number, 8.

*Tonsillitis.*—1842, November, 1 case. December, 3 cases. 1844, May, 1 case. 1845, January, 1 case. Total number, 6.

RESPIRATORY SYSTEM.

*Bronchitis.*—In March, 1843, 1 case.

*Catarrhus.**—1842, July, 54 cases. August, 35 cases. September, 12 cases. October, 23 cases. November, 32 cases. December, 21 cases. 1843, January, 16 cases. February, 30 cases. March, 34 cases. April, 27 cases. May, 51 csses. June, 92 cases. July, 16 cases. August, 6 cases. September, 9cases. October, 17 cases. November, 10 cases. December, 8 cases. 1844, January, 10 cases. February, 20 cases. March, 15cases. April, 14 cases. May, 11 cases. June, 6 cases. July, 6 cases. August, 19 cases. September, 5cases. October, 11 cases. November, 2 cases. December, 9 cases. 1845, January, 10 cases February, 15 cases March, 25 cases. April, 18 cases. May, 16 cases. June, 12 cases. Total number, 726.

*Hæmoptysis.*—1842, July, 1 case. 1844, March, 1 case. Total number, 2.

*Pthisis Pulmonalis.*—1842, December, 1 case. 1844, January, 1 case. Total number, 2.

*Pleuritis.*—1842, August, 4 cases. December, 1 case. 1843, March, 2 cases. April, 2 cases. May, 2 cases. September, 1 case. October, 1 case. 1844, January, 3 cases. February, 1 case. September, 1 case. October, 2 cases. November, 1 case. 1845, March, 2 cases. April, 4 cases. May, 1 case. June, 1 case. Total number, 29.

*Pneumonitis.*—1843, July, 1 case. September, 2 cases. December, 2 cases. 1844, January, 8 cases. 1845, February, 1 case. March, 1 case. April, 2 cases. May, 1 case. Total number, 18.

BRAIN AND NERVOUS SYSTEM.

*Apoplexia.*—1843, June, 1 case. July, 2 cases. Total number, 3.

*Cephalalgia.*—1842, August, 1 case. September, 1 case. November, 1 case. 1843, May, 2 cases. 1844, April, 1 case. May, 1 case. June, 1 case. 1845, March, 2 cases. May, 1 case. June, 1 case. Total number, 13.

*Delirium Tremens.*—1842, August, 2 cases. October, 2 cases. November, 2 cases. December, 4 cases. 1843, January, 2 cases. February, 2 cases. March, 3 cases. August, 1 case. 1844, March, 1 case.—April, 1 case. Total number, 20.

*Epilepsia.*—1842, October, 1 case. December, 1 case. 1843, January, 2 cases. February, 1 case. May, 1 case. June, 4 cases. July, 2 cases. August, 1 case. September, 1 case. December, 1 case. 1845, November, 2 cases. Total number, 17 cases.

*Paralysis.*—1844, February 1 case.

* The nosological distinction between catarrh and bronchitis is not readily defined: Writers and practitioners differ very much as respects the relative application of the two terms. It is probable that a large proportion of the cases included in these reports under the head of catarrh, would more commonly, and, perhaps, more appropriately be called cases of bronchitis.—EDITOR.

### URINARY AND GENITAL ORGANS.

As we can perceive no object to be answered in stating the months respectively in which the cases included in this division transpired, we give simply the total number for the whole period. EDITOR.

*Gonorrhœa*.—Number of cases, 73.

*Ischuria et Dysuria*.—Number of cases. 23.

*Orchitis*.—Number of cases, 18.

*Syphilis prim et secund*.—Number of cases, 56.

### FIBROUS AND MUSCULAR TISSUES.

*Rheumatism acut*.—1844, August, 1 case. November, 1 case. 1845, April, 1 case. Total number, 3.

*Rheumatism chron*.—1842, July, 7 cases. August, 2 cases. September, 5 cases. October, 4 cases. November, 9 cases. December, 2 cases. 1843, January, 3 cases. February, 5 cases, April, 2 cases. May, 3 cases. August, 1 case. September, 2 cases. October, 3 cases. November, 2 cases. December, 1 case. 1844, January, 2 cases. June 3 cases. July, 3 cases. August, 2 cases. September, 5 cases. October, 2 cases. December, 1 case. 1845, February, 1 case. March, 1 case. May, 1 case. Total number, 72.

As neuralgic affections have not a place in the table from which these cases are enumerated, we infer that the nosological classification prescribed to the medical officers of the army, does not include them under this head. We cannot speak definitely on this point, and have no means of obtaining, at once, information respecting it. If it be so, there is occasion to suppose that cases of neuralgia are embraced among those entitled Chronic Rheumatism. Neuralgia is an affection quite common in this locality; and the great number of the cases of chronic rheumatism in these reports, compared with those of the acute form of the disease, will, we think, surprise our readers, unless we adopt this explanation.—EDITOR.

### ABSCESSES AND ULCERS.

We give only the sum total of cases included in the remaining divisions.—EDITOR.

*Phlegmon et abscess*.—Total number, 4.

*Ulcers*.—Total number, 149.

### WOUNDS AND INJURIES.

*Am bustio*.—Total number, 7.

*Contusio*.—Total number, 8.

*Luxatio*.—Total number, 3.

*Vulnus incis*.—Total number, 58.

*V. Punct*.—Total number, 7.

*V. Sclopesticum*.—Total number, 1.

### ALL OTHER DISEASES.

*Amaurosis*.—Number of cases, 1.

*Debilitas*.—Number of cases, 5.

*Ebrietas*.—Number of cases, 47.

*Hæmorrhois*.—Number of cases, 11.

*Hernia*.—Number of cases, 10.

*Odontalgia*.—Number of cases, 14.

*Ophthalmia*.—Number of cases, 62.

*Otitis*.—Number of cases, 8.

*Prolapsus Ani*.—Number of cases, 2.

*Epistaxis.*—Number of cases, 2.

*Pernio*—Number of cases, 5.

*Morbi Varii.*—[Under this head are classed all diseases having no specific characters.—Ed.] Number of cases, 89.

NUMBER OF DEATHS.

Number of deaths during the whole period, 5.

NUMBER OF CASES.

Whole number of cases during the period, 2103.

TOTAL NUMBER OF OFFICERS AND MEN.

The number of officers and men for the months, respectively, of the period embraced in the reports is as follows: 1842, July, 314. August, 296. September, 284. October, 270. November, 264. December, 260. 1843, January, 260. February, 255. March, 250. April, 232. May, 220. June, 222. July, 232. August, 229. September, 224. October, 221. November, 231. December, 230. 1844, January, 231. February, 229. March, 228. April, 226. May, 228. June, 230. July, 230. August, 226. September, 231. October, 231. November, 229. December, 229. 1845, January, 229. February, 225. March, 229. April, 231. May, 231. June, 229.

---

Remarks.—It will be observed that the period of time embraced in the foregoing abstract is 3 years: the number of men in the command varying from 314 to 220, (12 com. officers, the remainder enlisted men,)—mostly adults, between the ages of 20 and 30 years; a few boys between 10 and 20 years of age.

These troops had been serving in the Florida war immediately previous to their arrival at this place, (about the last of June 1842,) different lengths of time, from one to five years. This will account for the high proportion of intermittents noted in the 3d and 4th quarters of 1842.

In the course of the 3 years some changes were made by discharges and enlistments; yet, as the number present in each month is stated, we have sufficient data to determine the ratio that the number of diseases bears to the number of men; and by making due allowance for the abiding effects of malaria, and of the exposure and wasting fatigue of field service in a hot, southern climate, we may, by a careful consideration of the foregoing abstract, form some estimate of the prevalent diseases incident to the several seasons of the year in this region of country. It will be noticed that (as would be anticipated) immediately after the change of climate, the number of intermittents rapidly decreased—there being 48 cases in July, 1842, 6 in January, 1843, and but 1 in April, 1843.—The predominance of Diarrhœa in the months of July, August and September, is constantly the same in each of the 3 years.

Diseases of the respiratory system seem to take precedence over all others even in the warm seasons—mark the great number of Catarrhs. Pleuritis appears to be a more common acute affection than Pneumonitis.

Rheumatism and Ophthalmia must be numbered among the most common complaints of this country. The small number of deaths—5 in 3 years, with an average number of 250 men—speaks well both of the climate, and of the care and skill of the attending Surgeon. These observations are made on taking a cursory glance ot the sbstraci; others will readily suggest themselves to the enlightened Physician.

Yours truly,

Dr. Flint. E. R. LONG.

*Buffalo, N. Y., July,* 1845.

---

We will only add to the preceding remarks, that the diminution in the number of cases of Delirium Tremens after the first quarter of the year 1843, may excite notice, and require some explanation. The fact is doubtless due to the influence of temperance principles which about this period became very generally diffused among the troops, and were, for the most part, inculcated, both by precept and example, by the officers. The same explanation, will, probably, apply to the affection next enumerated, viz: Epilepsy; which, it will be observed, diminishes in the frequency of its occurrence in the same proportion.—Editor.

---

### Letter from Accoucheur, No. 2.

Communicated for the Buffalo Medical Journal.

